# Clinically meaningful changes in functional independence among older patients with subacute stroke: estimating the minimal important change using an anchor-based adjusted predictive modeling approach

**DOI:** 10.1007/s41999-025-01401-x

**Published:** 2026-01-23

**Authors:** Hiroyuki Uchida, Tomoaki Shirakawa, Kazuki Ishii, Yudai Kato, Yuki Yamajo, Takumi Igusa, Masataka Sakimoto, Chihaya Machida, Tomohiro Shimada, Kenji Tsuchiya, Senichiro Kikuchi, Kazuki Hirao

**Affiliations:** 1 Graduate School of Health Sciences, Gunma University, Maebashi, Japan; 2 Department of Rehabilitation, Kurashiki Heisei Hospital, Kurashiki, Japan; 3 Department of Rehabilitation, Fujioka General Hospital, Fujioka, Japan; 4 Department of Rehabilitation, Medical Corporation Taiseikai, Uchida Hospital, Numata, Japan; 5 Department of Rehabilitation, Faculty of Health Sciences, Nagano University of Health and Medicine, Nagano, Japan; 6 Department of Occupational Therapy, School of Health Sciences, Faculty of Medicine, Kagoshima University, 8-35-1 Sakuragaoka, Kagoshima, 890-8544 Japan

**Keywords:** Minimal important change, Activities of daily living, Rehabilitation, Stroke, Functional independence measure

## Abstract

**Purpose:**

Functional independence measure (FIM) is a detailed assessment of activities of daily living in patients with stroke. To confirm that the FIM is useful in clinical practice and clinical research, the minimal important change (MIC), which is an aspect of interpretability of the FIM, must be clarified in addition to validity and reliability. This study aimed to estimate the MIC of the FIM in older patients with subacute stroke, with the goal of enhancing the interpretability of rehabilitation outcomes in geriatric care.

**Methods:**

Data from patients admitted to a single convalescent rehabilitation ward (CRW) in Japan between January 2020 and December 2022 were analyzed. This study included patients aged 65 years or older with subacute stroke who were originally living at home. Those who died or were transferred to other hospitals during hospitalization were excluded. FIM was assessed on admission and discharge. The MIC for the FIM was calculated using the place of discharge as an anchor (i.e., anchor-based adjusted predictive modeling method [MIC_adj_]).

**Results:**

Of the 1401 patients admitted to the CRW, 277 were eligible (mean age [SD], 78.9 [7.6] years). The motor, cognitive, and total FIM scores on CRW admission were 39.1 (21.2), 19.7 (8.5), and 58.8 (27.7) and those at CRW discharge were 60.9 (23.9), 23.9 (8.4), and 84.8 (30.7), respectively. The estimated MIC_adj_ for the motor, cognitive, and total FIM scores were 18.6, 3.9, and 22.8, respectively.

**Conclusions:**

The MIC for the FIM in older patients with subacute stroke were 19, 4, and 23 points for the motor, cognitive, and total FIM, respectively, which may aid in interpreting the effects of rehabilitation in older patients with subacute stroke.

## Key summary points

**Aim:**

This study aimed to estimate the minimal important change (MIC) of the functional independence measure (FIM) in older patients with subacute stroke, in order to enhance the interpretability of rehabilitation outcomes in geriatric care.

**Findings:**

The estimated MIC values for the FIM were 19 points for the motor domain, 4 points for the cognitive domain, and 23 points for the total score in older patients with subacute stroke.

**Message:**

These MIC thresholds may serve as clinically meaningful indicators for evaluating the effectiveness of rehabilitation interventions on activities of daily living in older patients with subacute stroke.

## Introduction

Stroke is a serious medical condition that requires long-term rehabilitation [1]. The incidence of stroke is currently increasing worldwide, and this increase is predicted to continue in the future [1]. Patients with stroke suffer from a range of problems, including paralysis, dysphagia, aphasia, and cognitive impairment, which negatively affect their health, resulting in a decline in their quality of life, onset of post-stroke depression, and increased medical expenses [1–5]. In addition, to these problems, the effect of strokes on activities of daily living (ADLs) is also an important issue that cannot be ignored [1, 6, 7].

Dependence on performing ADLs is common among patients with stroke. Many patients with stroke require some kind of assistance in performing ADLs such as grooming and walking outside [1, 7]. In addition, not only patients with severe strokes but also those with mild stroke experience ADL dependence [6, 8, 9]. In addition, considering that one of the rehabilitation goals for patients with stroke is to promote their discharge home and improving their ADL is important in achieving this, interventions to improve the ADL of patients with stroke are important [3, 10, 11].

At present, various rehabilitation strategies have been proposed to improve the ADL of patients with stroke [1, 12–14]. To assess the effectiveness of these intervention strategies, a scale that can appropriately evaluate interventions for ADLs in patients with stroke is crucial, as well as to examine its reliability and validity. Currently, several scales have been developed and are utilized to evaluate the ADL performance of patients with stroke [13–16]. Among these, the functional independence measure (FIM) stands out as one of the scales that is widely used scales globally to evaluate the ADL of patients with stroke [17, 18].

The FIM measures activity limitations in ADLs by observing the patient’s behavior and determining how much assistance is needed to perform basic physical and cognitive activities [17, 18]. Accordingly, the FIM is used as a standard for measuring changes in ADLs and quality of outcomes at the facility and national levels [16, 19–22]. However, to confirm the utility of FIM in clinical practice and clinical research, it is crucial to elucidate the minimal important change (MIC), which is an aspect of interpretability, in addition to validity and reliability.

The MIC is defined as the smallest score change that a patient or clinician would consider important among the measured components, and a score change that exceeds the MIC is interpreted as a clinically important change [23]. MIC decisions can help clinicians interpret changes in the outcomes and may enable them to modify interventions in clinical practice and propose more effective intervention strategies [23]. In addition, MIC plays an important role in determining the efficacy of treatment in clinical trials [23]. In clinical trials that utilize MIC as an evaluation index, the effect of the intervention is evaluated by comparing the proportion of patients who exceed the threshold determined by the MIC, and this method may be a more useful comparison method than the comparison of the mean values between groups [24]. Therefore, when evaluating treatment effects, the use of not only the mean value but also the MIC may help in interpreting the results of clinical trials. For these reasons, calculating the MIC for FIM holds significant importance in both research and clinical practice.

A previous study calculated the MIC for the FIM for patients with stroke [25]. However, this study may have included patients in the acute phase (within 6 days of onset) and subacute phase (7 days to 6 months after onset) of stroke in its registry; consequently, the estimated MIC for FIM may not be fully applicable to patients in the subacute phase of stroke [26]. In fact, a previous study has suggested that the estimated MIC may vary depending on the time elapsed since stroke onset [25]. Moreover, another study calculated the MIC of the FIM using the receiver operating characteristic (ROC) method rather than predictive modeling [25]. Although the ROC method is the most common approach for MIC calculation, it has been associated with several methodological problems; contrarily, predictive modeling is capable of addressing the issues associated with the ROC method, resulting in more accurate MIC calculations [23, 27]. Therefore, this study aimed to clarify the MIC of the FIM based on the predictive modeling method for patients with subacute stroke.

## Materials and methods

### Study design and setting

This longitudinal cohort study used routine evaluation data from patients admitted to the convalescent rehabilitation ward (CRW) at Kurashiki Heisei Hospital in Okayama Prefecture, Japan, between January 1, 2020, and December 31, 2022. In Japan, the CRW provides rehabilitation by a multidisciplinary team of physicians, nurses, physical therapists (PTs), occupational therapists (OTs), speech therapists (STs), etc., for up to 3 h a day, aiming for the return of inpatients to their homes [10]. The main targets are cerebrovascular disorders, neurological diseases, musculoskeletal diseases, and disuse syndrome that require ADL assistance during the subacute phase.

This study was approved by the Ethical Review Board for Medical Research Involving Human Subjects of Gunma University (Approval no. HS2024-067). Informed consent was obtained through the Kurashiki Heisei Hospital website using an opt-out system.

### Participants

The eligibility criteria for this study are as follows.

#### Inclusion criteria

(1) Subacute stroke patients (patients with cerebral infarction, cerebral hemorrhage, or subarachnoid hemorrhage) within 7 days to 6 months after onset [26, 28].

(2) Male and female.

(3) Age ≥ 65 years.

(4) Japanese native speakers.

(5) Individuals who originally lived at home.

#### Exclusion criteria

(1) Patients who died during hospitalization.

(2) Patients who were transferred to an acute care ward because of a change in condition.

In the present study, subacute stroke patients were defined according to the guidelines proposed by the Stroke Recovery and Rehabilitation Roundtable taskforce [26, 28]. This taskforce defines acute stroke as occurring within 6 days of onset and subacute stroke as occurring between 7 days and 6 months after onset [26, 28]. Therefore, in the present study, subacute stroke was defined as occurring between 7 days and 6 months after stroke onset. For patients who were hospitalized multiple times during the study period, only the data from the first hospitalization were used in the analysis.

### Measurements

Data were collected on admission and discharge from the CRW. All data were part of the routine clinical data collection and were extracted from the hospital information system, which include administrative data, clinical information, pharmacological data, and functional assessment scales. Sex, type of stroke, age, height, weight, body mass index, length of stay at the CRW, days from stroke onset to CRW admission, the FIM score at admission and discharge from the CRW, and place of discharge were collected.

#### FIM

The FIM measures activity limitations by observing the participant’s behavior and measuring the level of assistance required for basic physical and cognitive activities [17, 18]. The FIM is an 18-item ordinal scale that consists of 13 motor items (eating, grooming, bathing, dressing the upper body, dressing the lower body, toileting, bladder management, bowel management, transfer to bed/chair/wheelchair, transfer to toilet, transfer tub/shower, walk or wheelchair, and stairs) and 5 cognitive items (comprehension, expression, social interaction, problem solving, and memory). Each item is rated on a scale of 1–7 (1 = total assist, 2 = maximal assist, 3 = moderate assist, 4 = minimal assist, 5 = supervision, 6 = modified independence, 7 = complete independence). The total score ranged from 18 to 126 points (13–91 points for motor items and 5–35 for cognitive items), and a higher FIM score indicates that the patient requires less assistance and has fewer activity limitations. The reliability and validity of the FIM have been investigated in many studies [18, 29–32]. The FIM assessments were conducted by the therapist in charge of each patient at the time of admission and discharge. At Kurashiki Heisei Hospital, all therapists undergo regular training to ensure the quality of clinical data and prevent bias through participation in workshops and other means, in order to accurately conduct the FIM assessments. Furthermore, the inter-rater reliability of the FIM is considered good [32].

#### Place of discharge

The destination after discharge from the CRW (home or elsewhere) was used as the anchor. In many studies, self-administered rating scales such as the retrospective global ratings change (RGRC) and the global rating scale (GRS) are used as benchmarks when calculating the MIC [23]. However, the present study relied on past medical data, which were not obtained using these self-administered evaluation indicators. In such cases, objective evaluation indicators may be used as anchors [33, 34]. Contrarily, the objective anchors tend to strongly reflect the clinical perspectives and needs of the healthcare system [35, 36]. Therefore, to incorporate the patient’s perspective as much as possible, we considered that the destination at the time of discharge from CRW (home or elsewhere) would be an appropriate anchor. Previous studies have suggested that many older adults prefer to be discharged home [37, 38]. In addition, considering that one of the objectives of CRW in Japan is to improve the patients’ ability to perform ADLs and promote discharge to home, we considered that the discharge destination (i.e., discharge to home or elsewhere) would be an important anchor for calculating the MIC of the FIM, not only from a clinical perspective and in terms of the healthcare system needs, but also for patients themselves [10]. In fact, a previous study that calculated the MIC of the Barthel Index, a scale used to assess the patients’ ability to perform ADLs, employed home discharge at the time of discharge as a clinical anchor and regarded it as a clinically important improvement, as compared to not have been discharged home [39]. Thus, in the present study, the destination after discharge from the CRW (home or elsewhere) was used as the anchor.

### Rehabilitation programs

Older patients with subacute stroke admitted to the CRW received a rehabilitation program involving PTs, OTs, and STs under the direction of a doctor. These rehabilitation programs are performed for up to 3 h a day from the day of admission to the day of discharge. The rehabilitation program for older patients with subacute stroke in the CRW is designed based on the patient’s disease and individual goals and patient’s available physical, cognitive, and emotional resources to improve ADL and promote discharge to home. As a result, the intervention protocols varied; however, in general, PTs performed muscle strengthening exercises, static and dynamic balance exercises, transfer exercises, walking exercises, etc. OTs performed upper limb function and ADL exercises, and STs performed higher brain function and swallowing exercises.

### Sample size

Currently, the method for calculating the sample size for MIC has not been established yet [40]. However, previous study has suggested that a sample size of at least 100 is required to calculate the MIC [23]. The present research is a cohort study using past medical data. Therefore, although we did not perform a formal sample size calculation for this study, we concluded that collecting medical data from the CRW of Kurashiki Heisei Hospital from January 1, 2020, to December 31, 2022, would achieve a reasonable sample size (i.e., ≥ 100) for calculating the MIC of FIM in older patients with subacute stroke.

### Statistical analyses

All analyses were performed using R (version 4.4.2).

#### Minimal important change

The ROC method and the predictive modeling method, which are anchor-based approaches, were used to estimate the MIC [23]. In general, the predictive modeling method calculates a more accurate MIC (MIC_pred_) than the MIC (MIC_roc_) based on the ROC method. MIC_pred_ was calculated using the following formula, with logistic regression analysis centered on dichotomized anchors (home or elsewhere) as the dependent variable and FIM changes (changes from the time of CRW admission to the time of discharge) as the independent variable [23, 27]:

$$MIC_{pred} = \frac{{\ln \left( {odds_{pre} } \right) - C}}{{\mathrm{B}}}$$

$$odds_{pre} = \frac{Prevalence}{{1 - Prevalence}}$$

In the equation, C is the intercept calculated by logistic regression analysis, and B is the regression coefficient. odds_pre_ is calculated using the improvement rate of the anchor (discharge from the Hospital to home or otherwise). The predictive modeling method is suggested to introduce bias into the calculated MIC if the proportion of dichotomized anchors is not 50%. This bias will result in an overestimation of the MIC if the proportion of the improvement group is > 50% and an underestimation if it is < 50%. In particular, when the proportion of the improved group in the anchor exceeds 50%, the MIC is estimated to be higher; otherwise, it is estimated to be lower. Therefore, the MIC_adj_, which adjusted the bias of MIC_pre_, was calculated using the following formula [23, 27]:

$$MIC_{adj} = MIC_{pre} - S \times \ln \left( {odds_{pre} } \right)$$

$$S = 0.09 \times SD_{chang} + 0.103 \times SD_{chang} \times Cor$$

In this equation, Cor shows the correlation between the anchor and the change in the FIM, and SD_chang_ shows the standard deviation of the change in the FIM. Furthermore, the 95% confidence interval for the MIC was calculated using the bootstrap method (n = 2000) [23, 27].

#### Floor and ceiling effects

The presence of floor and ceiling effects influences the MIC [41, 42]. Therefore, the presence or absence of floor and ceiling effects was evaluated by examining the frequency of the highest and lowest scores at baseline. Floor effects were considered to exist when ≥ 15% of the patients had the lowest possible baseline score (13 points for motor, 5 for cognitive, and 18 points for total FIM) [41, 42]. The ceiling effect was considered to exist when ≥ 15% of the patients had a maximum baseline score (91 points for motor, 35 for cognitive, and 126 for total FIM) [41, 42].

## Results

### Participant characteristics

**Fig. 1:**
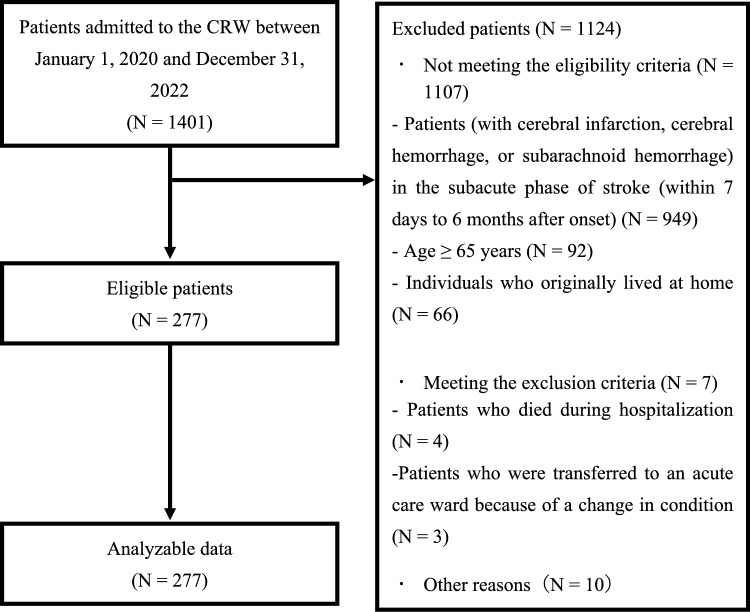
Flowchart of patients in the present study

Of the 1401 patients admitted to the CRW between 2020 and 2022, 277 met the eligibility criteria (Fig. 1). The demographic and basic clinical characteristics are shown in Table 1. The average (SD) age of the participants was 78.9 (7.6) years, and 137 (49.5%) were female. The mean scores at baseline for the motor, cognitive, and total FIM were 39.1 (21.2) points, 19.7 (8.5), and 58.8 (27.7), and those at the time of discharge were 60.9 (23.9), 23.9 (8.4), and 84.8 (30.7), respectively. In total, 187 (67.5%) patients were discharged to their homes, and 90 (32.5%) were discharged to other facilities (nursing homes, etc.).

**Table 1:** Characteristics of the participants

Characteristics	N = 277
Age (years)	78.9 (7.6)
Range	65–97
SEX	
Female	137 (49.5%)
Male	140 (50.5%)
Height	156.2 (9.2)
Range	131–184
Weight	52.6 (10.6)
Range	29.6–87.8
BMI	21.5 (3.5)
Range	13.4–33.3
Type of stroke	
Cerebral infarction	188 (67.9%)
Intracerebral hemorrhage	63 (22.7%)
Subarachnoid hemorrhage	26 (9.4%)
Days from stroke onset to CRW admission (days)	27.1 (16.7)
Range	7–114
Length of stay (days)	84.8 (45.2)
Range	9 –203
Discharge destination	
Home	187 (67.5%)
Other	90 (32.5%)
FIM at admission	
Motor	39.1 (21.2)
Range	13–89
Cognitive	19.7 (8.5)
Range	5–35
Total	58.8 (27.7)
Range	18–124
FIM at discharge	
Motor	60.9 (23.9)
Range	13–91
Cognitive	23.9 (8.4)
Range	5–35
Total	84.8 (30.7)
Range	18–126
Change FIM	
Motor	21.7 (14.5)
Range	−16–68
Cognitive	4.3 (4.8)
Range	−18–22
Total	26.0 (16.9)
Range	−26–84

Data are means (standard deviation) or numbers (%)

BMI: body mass index

CRW: convalescent rehabilitation ward

FIM: functional independence measure

#### MIC in all samples

The MIC_roc_, MIC_pred_, and MIC_adj_ for the motor, cognitive, and total FIM are shown in Table 2. The estimated values for the MIC_roc_ were 11.5 (95% CI 8.5–30.5) points for the motor, 4.5 (95% CI − 3.5 –5.5) for the cognitive, and 23.5 (95% CI 9.5–32.5) for the total FIM. The estimated MIC_pred_ values were 19.9 (95% CI 18.1–21.8) points on the motor, 4.2 (95% CI 3.6–4.9) points on the cognitive, and 24.2 (95% CI 22.0–26.2) points on the total FIM. The estimated values for the MIC_adj_ were 18.6 (95% CI 16.8–20.5) points for the motor, 3.9 (95% CI 3.2–4.6) for the cognitive, and 22.8 (95% CI 20.5–24.9) for the total FIM. The point-biserial correlation coefficients between the dichotomized MIC anchor and FIM changes were 0.29, 0.02, and 0.25 for the motor, cognitive, and total FIM, respectively.

**Table 2:** MIC estimation with the ROC and predictive modeling methods (N = 277)

	MIC_roc_	95% CI	MIC_pre_	95% CI	MIC_adj_	95% CI
Motor FIM	11.5	8.5–30.5	19.9	18.1–21.8	18.6	16.8–20.5
Cognitive FIM	4.5	-3.5–5.5	4.2	3.6–4.9	3.9	3.2–4.6
Total FIM	23.5	9.5–32.5	24.2	22.0–26.2	22.8	20.5–24.9

MIC: minimal important change

FIM: functional independence measure

### Floor and ceiling effects

No floor or ceiling effect was noted in any of the motor, cognitive, or total FIM.

## Discussion

This study provides important findings on the MIC of FIM. The MIC_roc_, MIC_pred_, and MIC_adj_ of the motor FIM were 11.5 (95% CI 8.5–30.5), 19.9 (95% CI 18.1–21.8), and 18.6 (95% CI 16.8–20.5). The MIC_roc_, MIC_pred_, and MIC_adj_ of the cognitive FIM were 4.5 (95% CI − 3.5– 5.5), 4.2 (95% CI 3.6–4.9), and 3.9 (95% CI 3.2–4.6), respectively. The MIC_roc_, MIC_pred_, and MIC_adj_ for the total FIM were 23.5 (95% CI 9.5–32.5), 24.2 (95% CI 22.0–26.2), and 22.8 (95% CI 20.5–24.9), respectively.

These emphasize that, as with existing MIC studies, the MIC estimates differ when different statistical approaches are used in FIM [43, 44]. In the present study, the MIC_pred_ values were the highest for motor and total FIM, and the MIC_roc_ value was the highest for cognitive FIM. Thus, the MIC_pred_ values for motor and total FIM and the MIC_roc_ values for cognitive FIM are likely to be the most conservative estimates. Conversely, simulation study in MIC estimation indicates that the ROC method is more susceptible to errors and has lower estimation accuracy than the predictive modeling method [27]. In fact, the 95% CI of MIC in the present study was narrower with the predictive modeling methods (i.e., MIC_pre_ and MIC_adj_) than that with the ROC method (MIC_roc_); these results are consistent with the findings of previous studies that estimated MIC based on the ROC and predictive modeling methods [43–45]. Furthermore, considering the advantages of the predictive modeling method, particularly its ability to adjust for bias when the proportion of improved patients is not 50%, the present study emphasizes that it is preferable to estimate the MIC of the FIM based on the predictive modeling method rather than based on the ROC method, and more specifically, to use the bias-adjusted predictive modeling method (i.e., MIC_adj_) [23, 27]. Therefore, because the MIC is expressed as an integer, it is highly likely that the MIC values for the motor, cognitive, and total FIM are 19, 4, and 23 points, respectively.

This study expands the evidence for the MIC study of FIM. The results of this study may help us accurately determine whether interventions for ADLs in older patients with subacute stroke are causing clinically meaningful responses. In addition, the MIC is useful not only in clinical settings but also for judging the effects of interventions in clinical research [23]. Considering that the FIM is widely used in clinical research, this score is likely to be valuable for researchers [12, 13, 24, 46]. In contrast, the MIC values calculated for motor (19 points), cognitive (4 points), and total FIM (23 points) were similar to those demonstrated in a previous study (17, 3, and 22 points, respectively) [25]. This indicates that the MIC for the FIM may not substantially differ between patients with acute or subacute stroke. However, the previous study included a mixed population of patients with acute and subacute stroke. Considering that the estimated MIC may vary depending on the time elapsed since stroke onset, future studies may need to estimate the MIC for the FIM by including only patients with acute stroke [25].

Furthermore, the similarity between the results of this study and those of a previous study may indicate additional advantages of the adjusted predictive modeling approach. Although point-biserial correlation coefficients of 0.3 or higher are suggested to be necessary for anchors and scale change points in appropriate MIC calculation, this study failed to meet that threshold for motor, cognitive, and total FIM [23]. However, a previous study suggests that the adjusted predictive modeling approach addresses the low point-biserial correlation coefficients between anchors and measurement change points; the results of this study may support this potential of the adjusted predictive modeling approach [47]. In fact, a previous study that calculated the MIC for the FIM in patients with hip fracture adopted a research design similar to that of the present study (discharge destination used as the anchor and the adjusted predictive modeling approach used for MIC calculation); although the point-biserial correlation coefficients between the anchor and the FIM change were low, the MIC values for the motor (24 points) and total FIM (26 points) were very close to those calculated using the ROC method (21 and 22 points, respectively) with an anchor showing good point-biserial correlation coefficients in another previous study [36, 48].

In addition, the adjusted predictive modeling approach may promote the use of a more valid objective anchor for MIC calculation. Previous studies have suggested the need to calculate the MIC using objective anchors, given that patient-reported anchors (e.g., RGRC and GRS) are not constantly appropriate and that objective anchors offer specific advantages, such as the ability to classify patients into improved and nonimproved groups, even among those with cognitive decline, unlike anchors relying on patient-reported assessments [33, 36, 49–52]. At present, although various objective anchors exist, indicators such as discharge destination and return to work are particularly important for patients, and the use of these indicators as anchors may result in the calculation of more clinically meaningful MICs that reflect the patients’ perspective [10, 25, 36, 39, 48, 53]. In contrast, factors such as discharge destination and employment maintenance are likely to be influenced by factors unrelated to functional improvement, and point-biserial correlation coefficients with outcome changes tend to be low [53]. However, the possibility of the adjusted predictive modeling approach to address these issues may strengthen the validity of using these objective anchors and provide new perspectives for future MIC research.

This study has several limitations. First, our study used clinical data collected during routine daily practice, which were not recorded as meticulously as data specifically obtained for research purposes. The potential for biases, such as data entry errors, cannot be ruled out [54]. Therefore, future studies must replicate the results of the present study based on a meticulously designed research plan, including data collection methods, to calculate the MIC of the FIM in older patients with subacute stroke. Second, this study used a clinical-based anchor (i.e., place of discharge) rather than a patient-reported outcome measure for calculating the MIC of FIM. Therefore, the estimated MIC may better reflect the needs of the clinical perspective and healthcare system than the expectations of patients. Future studies may need to calculate the MIC of the FIM using patient-reported outcome measures (i.e., RGRC and GRS) in older patients with subacute stroke. Third, data were obtained from a single medical institution in Japan. Thus, the results may not be representative of a wider population, and the generalizability of the results to other cohorts is unknown. Fourth, there was variation in the duration from admission to discharge for CRW patients in this study, as well as in the time from stroke onset to CRW admission. As many previous studies have used outcome changes from admission to discharge to calculate the MIC and the MIC of the FIM has been used to evaluate the rehabilitation effect on ADL performance from admission to discharge in patients with stroke, MIC calculation using FIM change from admission to discharge may result in a more accurate estimation of the intervention effect [25, 36, 55–57]. However, the variability in hospital stays among the patients may have influenced the MIC calculations. Similarly, differences in the time interval between stroke onset and CRW admission may have influenced the estimated MIC values. Therefore, future studies need to standardize the interval between baseline and follow-up assessments and recruit patients using stricter limits on the time from stroke onset to CRW admission to estimate the MIC of the FIM in older patients with subacute stroke.

## Conclusion

This study provided evidence regarding the MIC of the FIM in older patients with subacute stroke. The MIC values for the FIM in older patients with subacute stroke were 19, 4, and 23 points for the motor, cognitive, and total FIM, respectively, and these values may be important indicators for interpreting the effects of rehabilitation interventions on ADLs in older patients with subacute stroke. In the future, it will be essential to replicate the findings of this study using more appropriate anchors.

## CRediT authorship contribution statement

Conceptualization, H.U. and K.H.; methodology, H.U. and K.H.; formal analysis, H.U.; investigation, H.U., T.S., K.I., Y.K. and Y.Y.; resources, H.U. and K.H.; data curation, H.U.; writing—original draft preparation, H.U. and K.H.; writing—review and editing, H.U., T.S., K.I., Y.K., Y.Y., T.I., M.S., C.M., T.S., K.T., S.K. and K.H.; project administration, H.U.; supervision, K.H. All the authors have read and agreed to the published version of the manuscript.

## Data Availability

The datasets analyzed in this study are not publicly available due to ethical and privacy reasons, but are available from the corresponding author upon reasonable request.
